# HPV-DNA testing for cervical cancer precursors: from evidence to clinical practice

**DOI:** 10.3332/ecancer.2012.258

**Published:** 2012-06-18

**Authors:** M Origoni, P Cristoforoni, S Costa, L Mariani, P Scirpa, A Lorincz, M Sideri

**Affiliations:** 1 Department of Obstetrics & Gynecology, School of Medicine, Vita-Salute San Raffaele University, Via Olgettina 60, 20132 Milano, Italy; 2 Gynecologic Oncology Unit, National Institute on Cancer Research (IST), Genova, Italy; 3 Obstetrics & Gynecology Unit, “Policlinico S. Orsola-Malpighi” University Hospital, Bologna, Italy; 4 Gynecologic Oncology Unit, “Regina Elena” National Cancer Institute, Rome, Italy; 5 Department of Obstetrics & Gynecology, School of Medicine, Catholic University of the Sacred Heart, Rome, Italy; 6 Wolfson Institute of Preventive Medicine, Queen Mary University of London, UK; 7 Preventive Gynecology Unit, European Institute of Oncology, Milano, Italy

**Keywords:** cervical cancer, human papillomavirus (HPV), HPV-DNA, screening

## Abstract

The large amount of literature published over the last two decades on human papillomavirus (HPV)-DNA testing has definitely demonstrated the association between high-risk viral genotypes (hrHPV) and cervical cancer. Moreover, hrHPV-DNA testing has shown excellent performance in several clinical applications, from screening settings to the follow-up of treated patients, compared to conventional cytology or colposcopy options. On the other hand, when a huge number of reports are published on the same subject in a relatively short period of time, with many variations in settings, study designs and applications, the result is often confusion and decreased comprehension by readers. In daily office practice, several different situations (in symptomatic or asymptomatic women) can be positively managed by the correct use of hrHPV-DNA testing. Validated hrHPV-DNA testing and, specifically, the HC2® assay, due to its excellent sensitivity and negative predictive value together with optimal reproducibility, currently represent a powerful tool in the clinician’s hands to optimally manage several situations related to HPV infection and the potential development of cervical cancer.

## Introduction

Despite the large amount of data available on the value of human papillomavirus (HPV)-DNA testing for the detection of cervical cancer precursors, both in the primary cervical screening and in the management of ‘borderline’ or ASCUS (Atypical Squamous Cells of Undetermined Significance) cytology, HPV-DNA tests have not always and correctly been translated into clinical practice by clinicians and within national cervical screening programs. This article is intended as a synthesis of the literature aimed at supplying useful and simplified ‘take-home messages’ to the clinical community. The recommendations have been formulated consistent with the evidence-based literature and rated according to their level of quality and strength, employing a generally recognized and approved rating system, described in Table 1 [1–3].

## HPV testing in primary screening

Several population-based studies have established that tests for high-risk HPV (hrHPV) DNA have higher clinical sensitivity than cytology in detecting cervical intraepithelial neoplasia (CIN) of grades 2 and above (CIN2+) [4, 5], and that combined HPV and cytology testing shows the highest negative predictive values (NPV) for CIN2+ [6–8]. The randomized trials found that while some of the CIN2 may spontaneously regress, the increased sensitivity for precancers and cancers, grouped here as CIN3+, is not merely an overdiagnosis, as there is a corresponding lower incidence of future CIN3+ [9–12].

Increased sensitivity has two important clinical outcomes: reduced mortality and an elongation of screening intervals; the latter implies better compliance with screening and lower costs. Recently, two papers confirmed these early assumptions. A publication from rural India, a setting where no population screening is in place, showed that a single round of hrHPV testing by Hybrid Capture 2 (HC2®) was associated with a significant decline in the rate of advanced cervical cancers and associated deaths, as compared with the unscreened control group [13]. In contrast, in the same study there was no significant reduction in the rate of death in either the cytology-testing group or the VIA (visualization of the cervix after the application of acetic acid) group. The second paper reported the final results of a large population study from Italy; the study showed that HPV-based screening is more effective than cytology in preventing invasive cervical cancer, by detecting persistent high-grade lesions earlier and providing a longer low-risk period. The detection of invasive cervical cancers was similar for the two groups in the first round of screening (nine in the cytology group vs. seven in the HPV group, p = 0.62); no cases were detected in the HPV group during Round 2, compared with nine in the cytology group (p = 0.004). Overall, in the two rounds of screening, 18 invasive cancers were detected in the cytology group vs. seven in the HPV group (p = 0.028) [14]. Finally, a recent meta-analysis of seven European population studies published in BMJ showed that primary screening by HPV testing allows for an increase in the interval between two screenings of up to six years, while keeping the cumulative incidence rate of CIN3+ in HPV test negative women almost two times lower (0.27%) than in women screened by cytology (0.51%) at three year intervals [15]. The authors suggested that cervical screening strategies in which women are screened for HPV every six years are as safe and effective as three-yearly cytology screening. In this way, even an increase of costs related to the new technology is balanced by an extension of intervals between two different screens. Interestingly, the most obvious source of heterogeneity between the different European countries was the variability in interpretation of cervical smear tests, as the proportions of positive cytology results ranged from 2% in Sweden to 7% in France.

Another added value of HPV testing over cytology is its high reproducibility: Carozzi [16] investigated the intra- and inter-laboratory reproducibility from a large Italian population study (NTCC) involving seven laboratories with different levels of experience and confirmed the high reliability and reproducibility of the HC2 assay at each site.

While the NPV of HPV testing over the Pap smear is well established, the management of HPV positive patients in the screening setting is still controversial. In a recent paper, different strategies have been evaluated: the authors concluded that primary HPV-DNA-based screening with cytology triage and repeat HPV-DNA testing of cytology-negative women appears to be the most feasible strategy [17]. The American Society for Colposcopy and Cervical Pathology (ASCCP) proposed HPV genotyping to triage women who are HPV screening test positive but cytology negative; in this group of patients, an immediate colposcopy is indicated if genotyping is positive for HPV 16 and/or 18 [1, 18]. A large prospective Italian clinical trial (NTCC) investigated the determination of p16^INK4A^ (P16) to triage positive women initially screened with HC2®; this strategy showed the same positive predictive value of conventional Pap smear screening (no substantial increase in referral to colposcopy), while retaining the higher NPV of screening by HPV testing [19]. In the USA, the American Cancer Society (ACS) and ASCCP guidelines mention the option to use a combination of cytology and HPV-DNA testing [1, 20]. A screening model combining both tests offers the highest NPV; however, compared with HPV testing alone, the small increase in sensitivity is counterbalanced by higher costs due to the need for twice as many screening tests [15].

According to the emerging scenario of the post-HPV vaccination era, it is to mention the particular value of hrHPV-DNA testing as the most suitable screening test for vaccinated women; what is expected from vaccination in the next future, a very low prevalence of HPV-correlated diseases, depicts another added value of this screening option.

Conclusions about the use of hrHPV-DNA testing in primary screening are summarized in Table 2.

## Management of ‘borderline’ cervical cytology

Pap test reporting classifications have evolved during the last decades, with the current standard being the Bethesda System in its latest 2001 revision [21, 22]. Of the estimated 50 million Pap smears performed each year in the United States, more than 5% are reported as abnormal. A comprehensive study demonstrated that ASCUS cytology reports, by their nature, are not highly reproducible, even among expert cervical cytopathologists [23]. The risk of invasive cancer in patients with ASCUS cytology is low, ranging from 0.1 to 0.2%. Nevertheless, from 5 to 17% of patients with ASCUS and from 24 to 94 % of those with ASC-H (atypical squamous cells suggestive of high-grade squamous intraepithelial lesion; HSIL) will have CIN2+ at a colposcopically directed biopsy [24]. From a clinical point of view, the goal is to identify the minority of women with underlying CIN2+ among the large numbers of ASCUS and low-grade squamous intraepithelial lesions (LSIL), avoiding excessively aggressive triage.

In the USA, the vast majority of women diagnosed with an ASCUS are triaged to colposcopy or other follow-up by an hrHPV-DNA test. In contrast, outside the USA there has been a long lasting lack of consensus as to the appropriate management of the millions of women with ASCUS or equivocal cytologic abnormalities. Three major management strategies have been used in Europe. Immediate colposcopy has been assumed to be the safest option but with the disadvantages of high costs and potential overtreatments. Because the sensitivity of a single round of conventional cervical cytology is relatively low, a program of repeated cytology has been used in most countries. Several large studies have evaluated the performance of hrHPV-DNA testing to guide management in the ASCUS population, with sensitivity for the detection of CIN2+ using HC2® reported as 83–100% [25].

The ASCUS/LSIL Triage Study (ALTS) represents a hallmark randomized, multicenter clinical trial sponsored by the National Cancer Institute designed to compare three management options [26]. HPV-DNA testing (HC2®) showed the highest sensitivity and identified 96.3% (95% CI 91.6–98.8) of women with CIN3+ [27]. Several recently published studies also support the concept that high-risk HPV-DNA testing offers an effective triage method for women with equivocal cytologic abnormalities [28, 29]. High sensitivity, combined with a reasonable specificity for triage, makes HPV-DNA testing the recommended option for the management of ASCUS cytology (AI). The ALTS study also provided longitudinal data by following women with an original report of ASCUS every six months over a period of twoyears. These data, published in 2003, demonstrate that the HPV-DNA testing option is at least as sensitive as an immediate colposcopy for detecting CIN3+ among women with ASCUS and suggest that the HPV-DNA testing triage is the most effective strategy for the management of women with ASCUS (AI). A follow-up by repeated cytology, with colposcopic referral at an ASCUS threshold, is also sensitive in detecting CIN3+ but requires repeated visits and leads to significantly more colposcopic examinations than does a single HPV-DNA test (AI). The immediate colposcopy strategy is certainly the least specific (AI) [30].

Based on these studies, the ASCCP, in the 2006 consensus guidelines for the management of women with abnormal cervical cancer screening tests, recommends a HPV-DNA testing-based triage for women with ASCUS cytology [1]. Cervical cytologic testing or colposcopy is a acceptable method for managing women over the age of 20 years with ASCUS, but HPV-DNA testing is the preferred approach (AI). Women with ASCUS who are HPV-DNA negative can be followed up with repeat cytologic testing at 12 months (BII). Women who are HPV-DNA positive should be managed as women with LSIL and be referred for a colposcopic evaluation (AII). HPV-DNA testing should not be performed at intervals of less than 12 months (EIII).

Evidence-based statements about the ASCUS cytology management are summarized in Table 3.

## Follow-up of treated CIN2+

An important setting where hrHPV-DNA testing has shown potential benefit is the follow-up of women treated for CIN, to detect residual or recurrent disease [31]. The long-term risk of subsequent high-grade CIN (CIN2+) and invasive cervical carcinoma remains higher among women treated for CIN, and they require continued surveillance and follow-up [32, 33]. Moreover, it has been observed that residual or recurrent disease in women with persistent HPV 16 and/or HPV 18 is higher (82%) than in women with persistence of other hrHPV types such as HPV 31, 33, 35, 45, 52 and 58 (66.7%) or HPV 39, 51, 56, 59, 68, 26, 53, 66, 73 and 82 (14.3%). These data suggest different risk levels for the progression of CIN. Hence, the detection of a persistent infection with certain hrHPV genotypes has the potential to improve patient management [34]. To identify factors that may predict long-term results of CIN treatment and HPV clearance/persistence after conservative excisional therapy, Costa [31] analysed a series of 252 women with CIN treated by electro-surgical conization (EC) using sequential hrHPV-DNA testing during post-treatment follow-up. The hrHPV-DNA positivity was reduced from 90 to 20% (p < 0.01) at the second visit after EC, but HPV persistence was strongly correlated with residual/recurrent CIN. Significant independent predicting factors of HPV persistence according to a multivariate Cox model are listed in Table 4. In their recent meta-analysis, Arbyn et al [35] analysed the studies in which the HC2 assay was used in patients after treatment of CIN for monitoring purposes. Treatment failure expressed in terms of residual or recurrent CIN occurred in 10.2% (95% CI 6.7–13.8) of treated cases. Despite data heterogeneity, the pooled hrHPV-DNA results after treatment predicted residual/recurrent CIN with a significantly higher sensitivity (odds ratio (OR) = 1.27; 95% CI 1.06–1.51) and a non-significantly lower specificity (OR = 0.94, 95% CI 0.87–1.01) than conventional cytology-based follow-up. Overall, combined hrHPV and cytology testing yielded the best performance; the combination hrHPV-DNA testing and cytology demonstrated a 96% sensitivity (95% CI 89–99), 81% specificity (95% CI 77–84), 46% PPV (95% CI 38–54) and 99% NPV (95% CI 98–100) [35]. Additional studies comparing the performance of the combination of the HC2® assay and Pap smear in detecting residual and recurrent disease as well as hrHPV persistence after treatment of high-grade CIN showed a specificity and PPV significantly higher than those of the single tests alone [36, 37].

In conclusion, for women double negative (both Pap and HC2® tests) at six months retesting at 12 months could be safely skipped, maintaining the 24-month follow-up visit scheduled [37]. In studies where lesions were treated by excision, hrHPV-DNA testing significantly predicted treatment outcome with higher sensitivity and higher specificity than the pathological assessment of the cone margins: relative sensitivity 1.31 (95% CI 1.11–1.55) and relative specificity 1.05 (95% CI 0.96–1.15). Overall, combined hrHPV testing and cytology yielded the best test characteristics (AII).

It can therefore be concluded that the evidence-based post-treatment follow-up should include both the conventional cytology and HC2® testing to identify patients at increased risk for disease recurrence (AII).

## Glandular and adenocarcinoma in situ (AIS) lesions

Over the past 50 years, the relative proportion, as well as absolute incidence, of invasive and pre-invasive glandular lesions of the uterine cervix has been increasing in western countries. Reports from the 1950s and 1960s indicated that adenocarcinomas accounted for only 5% of cervical cancer cases, while in the 1970s they represented 20–25% of all cervical carcinomas [38]. In recent years, conservative surgery has been proposed because the majority of cases are in young fertile women. Nevertheless, the management of Adenocarcinoma in situ (AIS) continues to be a controversial issue [39, 40]. In a review of 14 studies comprising 157 AIS patients with negative conization margins, 26% harboured residual AIS and in 2% a unsuspected invasive cancer was disclosed, implicating hysterectomy as definitive treatment [41]. The conservative alternative to a hysterectomy is conization with close surveillance, conventionally performed by repeat cytology, colposcopy and, eventually, punch biopsy and endocervical curettage. Unfortunately, these methods have a substantial false negative rate for glandular lesions both in primary diagnosis and in the follow-up of treated patients [41, 42]. Recent results showed that HC2® predicted residual/recurrent AIS or invasive Adenocarcinoma (AdCa) during the follow-up of conservatively treated women for AIS significantly better than cytology, whose predictive power did not reach statistical significance at any of the FU visits. Furthermore, HC2® showed a better NPV, while cytology was more specific in detecting residual AIS (AIII). The combination of HC2® and cytology reached 90% sensitivity in detecting persistent lesions at the first FU visit and 100% sensitivity at the second FU visit [42] (Table 5).

In conclusion, in the follow-up of AIS patients who desire to preserve fertility, the combination of HC2® and cytology showed a high sensitivity in detecting persistent lesions. In addition, the NPV of 100% seems to be very useful in preventing unnecessary hysterectomies (AIII) (Table 6).

## HPV genotyping

More than 40 different genotypes of HPV can cause infections of the cervix. Based on available cross-sectional and short-term prospective data, the risks of persistence and neoplastic progression markedly differ by HPV type [43, 44] but most of them are extremely rare and therefore not included in screening tests. HC2® has 13 HPV types in a carcinogenic probe cocktail, and these types are responsible for the vast majority of cervical cancers. The clinical management algorithm includes as many as four or five options (treatment, colposcopy and directed biopsy, follow up at six months, 12 months or regular screening), so that individual genotyping test results (there are more than 13 different possible results even without including multiple infections) cannot be used to create a meaningful clinical management protocol. The risk stratification algorithm still lacks sufficient clinical data to be fully implemented in the clinical setting [45]. The performance of different genotyping tests can vary greatly from test to test and among different laboratories, lowering reproducibility; in addition genotyping is carried out by PCR-based methods, many of which show excessively high-analytical sensitivity, detecting clinically irrelevant infections. High-analytical sensitivity therefore lowers the positive predictive value of the test and potentially generates substantial harm to the patient.

Several studies investigated the potential use and limitations of hrHPV genotyping in screening, the triage of borderline smears and post-treatment follow-up clinical situations. At present, only the ASCCP guideline includes genotyping in the management algorithm for use in the screening setting of triage cytology negative and hrHPV-DNA positive patients. HPV 16 and/or 18 positive women are recommended for immediate colposcopy, while HPV 16 and/or 18 negative but other hrHPV positive women are referred to annual follow-up visits. The clinical evidence supporting this clinical guideline is supported by many studies [18] (AII).

HPV 16 and/or 18 positive women with an ASCUS or low-grade SIL Pap smear carry an increased risk of CIN2+ lesions. In the ALTS study, women with ASCUS or LSIL who were HPV16 positive were at increased risk for ≥CIN3 compared with women who were HPV negative (OR = 38; 95% CI 22–68; p < 0.001); this risk was fivefold greater than the risk in women who were positive for non-HPV 16 carcinogenic HPV types (OR = 7.2; 95% CI 4.2–13; p < 0.001) [46] (CII). These observations led to the suggestion that the triage of borderline smears treatment should be instituted on the basis of a positive Pap smear and genotype result only. However, at present, this is still a suggestion, as there is not enough evidence to change the clinical practice.

HPV-DNA testing has been introduced into clinical practice as a cure test. Some studies investigated the potential use of genotyping in this clinical setting [34, 47]. There is strong evidence that genotype-specific persistence predicts short-term recurrence and that, conversely, clearance of the HPV genotype associated with a preoperative diagnosis implies successful treatment and a low risk of recurrence; however, this clinical algorithm still needs to be verified (CII).

At present, there are no clear indications of the appropriate use of genotyping in clinical practice; testing without changing the clinical protocol carries the potential to increase costs and harm to the patient, with no added benefit. It is recommended that the performance of the many HPV genotyping tests available on the market should be clinically validated by substantial clinical data and acceptable test inter-laboratory reproducibility confirmed before implementing genotyping into clinical practice. Due to current test limitations, the clinical application of genotyping is not included in most international guidelines.

## Cost-benefit analysis of HPV-based screening

Cytology has reduced the incidence and mortality of cervical cancer in countries with organized screening, but in Europe there are still an estimated 68,000 incident cases while in the USA there are ~12,000 new cases per year [48]. Nevertheless, because of its relatively limited sensitivity, requiring frequent repetition of the test and a high level of organization, cytology-based screening leads to a high-economic burden.

The impending broad implementation of cervical cancer screening by hrHPV-DNA testing as a first-line screen should take into account both the substantial impacts of ongoing vaccination programs, with two vaccines showing efficacy in preventing both infections and associated precancerous lesions [49, 50] due to HPV 16 and 18, which are responsible for about 70% of cervical cancer cases, and the clinical consequences of such intervention over the coming years (i.e. reduction of HPV prevalence). Policy makers need qualitative and quantitative insights regarding the realistic potential consequences of new screening technologies. Nevertheless, the clinical outcome of any new hrHPV-based screening strategies (documented as population-wide reductions in mortality from cervical cancer) will not be clearly observable for decades and, inevitably, decision-making must rely on studies reporting intermediate outcomes and mathematical models ensuring consistency with epidemiologic observations.

Multiple variables simulating the natural history of cervical cancer, and specific details and costs of delivering clinical interventions, are used in such complex modelling systems. Moreover, models have to be tailored to the specific populations targeted for screening programs, which may significantly differ in HPV infection prevalence, rates of other related cervical cancer risk factors, costs and cost-related variables. The rate of screening coverage and adherence radically impacts each given scenario.

Although well-designed model-based simulations are considered to be useful and predictive for future clinical applications, real world validity should be further confirmed. As recently stated by the European Guidelines for Quality Assurance in Cervical Cancer Screening, a *posteriori* analysis of observed data, as obtained through continued surveillance and post-marketing studies, is mandatory [51].

The most relevant variables used by mathematical models to evaluate the cost-efficacy of hrHPV-DNA testing-based screening are the longitudinal clinical performance indicators: sensitivity, specificity and positive-NPVs. Regarding clinical sensitivity, a recent meta-analysis reported a pooled sensitivity for HC2® in primary screening of 97.9% (AI) [5]. The expected loss in specificity with hrHPV-DNA testing in women aged 35 years or more is relatively small: 93.3% [4]. The NPV of hrHPV testing is extremely high as confirmed in several studies [52–55].

Considering the epidemiology and natural history of HPV infections, selecting the appropriate age of screening is important. The age of 30 years (concerning the age-related decline of transient infections) has been considered appropriate to start hrHPV-DNA screening in Europe [56, 57] (AIII). Primary hrHPV-DNA screening of women younger than 30 years old is not recommended due to the high rate of HPV-DNA testing positivity and low specificity, implying a detection of a large amount of regressive lesions (AIII), as recently stated by the NTCC study [14].

Considering that HC2® is at present more expensive and less specific than conventional cytology, hrHPV-DNA-based screening must have the added value of allowing a reduced screening frequency. This is possible and safe because multiple large clinical trials have consistently shown a very high-NPV in women having a negative baseline HC2® result. Kim et al. [57] showed that in the context of four European countries (United Kingdom, Netherlands, France and Italy)—using a complex cost-efficacy mathematical model—the most effective strategy is the combination test (Pap test and hrHPV-DNA over the age of 30) within a three year screening interval. Nevertheless, the already quoted Finnish and Italian trials showed, in women over 35 years of age, a higher efficacy of primary cervical hrHPV-DNA testing followed by cytologic triage compared conventional cytological screening [14, 58]. Furthermore, in the German healthcare context, the introduction of hrHPV-DNA-based screening strategies is a cost-effective alternative to cytology when compared to any screening interval longer than one year [59].

If HPV vaccination is included in the analysis, the following strategies were valued as being more cost-effective than the ongoing cytology screening:

- unvaccinated women: screening starting at 25 years with cytology with hrHPV-DNA testing triage and switching to hrHPV-DNA testing with cytology triage at 30 years (rounds every three years);
- vaccinated girls (vaccination starting at age 12 years): screening starting at 25 years with hrHPV-DNA testing with cytology triage (rounds every five years) [60].

The optimal screening strategy for women vaccinated at older ages (18 years or above) has not yet been determined, and until specific guidance data are available, they should be screened as per the algorithm for unvaccinated women.

A cost-effectiveness analysis in 2006 concluded that hrHPV-DNA testing is an economically viable strategy for the triage of ASCUS cytology (AI) [61].

## Counselling

The proper education of women and of the various healthcare professionals involved in the process are fundamental aspects in the area of HPV-DNA testing. The fact that cervical carcinoma is caused by a virus which is sexually transmitted introduces additional complexity and high-emotional impact to the doctor–patient relationship. HPV is extremely widespread in healthy populations, and the concepts of temporary infection, persistent infection, and illness are strikingly different, but conceptually difficult to understand. The availability of the HPV test in primary screening implicates the management—clinical and psychological—of a fair number of women with normal cytology and positive viral tests, especially if young women (<30 years old) are included. Resorting to self-obtained information in the area of health is in continuous expansion, and frequently, the sources of the available materials are of debatable quality. HPV and related diseases are certainly newsworthy and of interest to the media.

The minimum set of information for women is summarized in Table 7.

Available time, adequacy of the environmental setting, technical competence, the communication skills of the health professional and the receptive level of the patient are the most important variables to consider and possibly to face [60, 62–64]. Great benefit in daily practice can be gained by the availability and use of quality information (scientifically correct, extremely concise and comprehensible in content and in display, graphically attention getting) provided in a timely manner. The best time to provide information on HPV is in fact before performing the tests: at this point, the level of patient attention and capacity of comprehension are high and anxiety quite low.

The whole universe of cervical pathology should, in our minds, be framed in the concept of cervical cancer risk. Considering specifically the persistent positivity of viral tests as one of the most significant indicators in the degree of probability that a woman, over time, will develop a serious cervical pathology—as is similarly done in cases of plasma cholesterol levels or obesity in cardio-vascular risk—may help to correctly position the examination in the imaginary collective and introduce implications of varying follow-up procedures (i.e., repetition of the test, Pap smear, colposcopy, viral typing and other biological markers) in relation to risk stratification [65].

In light of this, the positivity of the test does not represent an illness, but simply the presence of a risk factor that, if confirmed one year after the first sampling, will suggest the plain observance of a non-invasive cytological or instrumental triage. As a whole, first and second level cervical screening tests guarantee a degree of protection from the real disease—invasive carcinoma—that has no equivalent in medicine (AI).

## Discussion

Human papillomavirus (HPV) is well-recognized as the necessary cause of neoplastic transformation of the uterine cervix epithelia. As regards uterine cervical cancer prevention, a significant body of literature has definitely demonstrated the higher performance of hrHPV-DNA testing over cytology in terms of sensitivity, negative predictive power (NPP) and reproducibility in different clinical settings: primary screening, triage of ‘borderline’ (ASCUS) cytology and follow-up of treated preneoplastic disease (CIN). Compared to cytology, either conventional or liquid based, internationally validated HPV-DNA tests demonstrate a very close to 100% sensitivity and NPV in the identification of cervical preneoplastic conditions. Thus, the new screening algorithm should include hrHPV testing as primary test, with cytological triage only in hrHPV positive women. However, although the validity of hrHPV-DNA testing is already worldwide accepted as triage for ASCUS cytology or as test of cure after CIN therapy, it is not yet translated as front-line test into clinical practice in organized programs (with the exception of the Netherlands) or in opportunistic screening as well. The added value, compared to conventional cytological screening, is concerning not only squamous cervical lesions, but also glandular types (adenocarcinoma in situ, AIS), which are less prevented by the national programs of surveillance.

In view of the epidemiology and natural history of HPV infections, the age of 30 years appears to be appropriate to start hrHPV-DNA testing as primary screening, avoiding younger women where HPV-infection is highly prevalent, consisting in large amounts of spontaneously regressive lesions.

Due to variability in performance (low reproducibility and excessively high-analytical sensitivity), no clear indications of the appropriate use of HPV genotyping in clinical practice can be drawn. Unfortunately, the huge amount of data of literature in the field is not consistent with the clinical practice, and many clinicians still rely on Pap smear or suggest to patients the use of HPV-DNA testing with questionable indications; strong evidence-based recommendations can be made in order to achieve the best results of cervical cancer prevention: primary screening, triage of ASCUS cytology and follow up of conservatively treated intraepithelial cervical lesion are definitely the settings in which HPV-DNA testing is the optimal option.

## Figures and Tables

**Table 1:** Evidence-based rating system for clinical recommendations

*Recommendation ratings*	
A	Good evidence for efficacy and substantial clinical benefit support recommendation for use.
B	Moderate evidence for efficacy or only limited clinical benefit supports recommendation for use.
C	Evidence for efficacy is insufficient to support a recommendation for or against use, but recommendations may be made on other grounds.
D	Moderate evidence for lack of efficacy or for adverse outcome supports a recommendation against use.
E	Good evidence for lack of efficacy or for adverse outcome supports a recommendation against use.
*Quality of evidence*	
I	Evidence from at least one randomized, controlled trial.
II	Evidence from at least one clinical trial without randomization, from cohort or case-controlled analytic studies (preferably from more than one center) or from multiple time-series studies or dramatic results from uncontrolled experiments.
III	Evidence from opinions of respected authorities based on clinical experience, descriptive studies, or reports of expert committees.

**Table 2:** Evidence-based statements about the use of HPV test in primary screening

1	High-risk HPV DNA testing alone, as a primary screening method, has been shown to be more sensitive than cytology in several clinical studies (AI).
2	HC2 proved to be superior to Pap smear screening in reducing cervical cancer mortality in one trial in rural India (AI).
3	The negative predictive value of HR HPV DNA testing every 6 years was higher than the negative predictive value of Pap smear screening every 3 years in a recent metanalysis; six of the seven studies analyzed used HC2 as the HPV testing method (AI).
4	It is appropriate to begin large scale demonstration projects using HPV screening as the sole primary screening test in patients older than 30 years (AIII).
5	Efficient management of women who are HPV positive based on a single screening test remains a key issue for primary screening. Cytologic triage of HPV positive women with retesting for HPV at one year (or possibly two years) for those who are HPV positive and cytology negative appears to be the most feasible option (AI). Other options include: HPV genotyping for types 16, 18 and possibly 45 (AII) or triage using P16 on the cytology slide prepared from the original HPV testing fluid (AI).
6	Co-testing (simultaneous cytology and HPV testing) offers the highest negative predictive value at a cost of more than double the tests needed; however, double testing does not significantly affect the positive predictive value of single testing (no significant increase in colposcopic examinations) (AI).
7	HPV testing is superior to cervical cytology in inter-laboratory and inter-observer variability (AI). However it is important that HPV testing be conducted with a clinically validated test to ensure that results are objective and highly reproducible.
8	HC2 is clinically validated in population screening (AI) and shows high inter and intra-laboratory reproducibility (AII).
9	HPV testing is the most sensitive and specific screening test in the post vaccination (AIII).

**Table 3:** Evidence-based statements about ASCUS cytology management.

(a)	HR HPV-DNA testing is the most effective strategy (AI).
(b)	HR HPV-DNA testing is cost-effective (AI).
(c)	HC2 is the most widely validated test (AI).
(d)	HC2 - negative patients can be referred to screening (BII).
HC2: Hybrid Capture 2	

**Table 4:** Independent predicting factors of HPV persistence after conservative excisional therapy for CIN2+

Independent predictors (Cox model)	*P* value
Positive endocervical margin	0.001
Lesion grade in the cone	0.004
High-grade lesion in the colposcopic biopsy	0.023
Age above 35 years	0.029
HSIL in Pap smear	0.029
Cigarette smoking	0.029

**Table 5:** Performance of high-risk HPV-DNA testing + cytology in predicting residual/recurrent CIN [5, 6]

Sensitivity	96% (95% CI 89–99)
Specificity	81% (95% CI 77–84)
PPV	46% (95% CI 38–54)
NPV	99% (95% CI 98–100)
*PPV* positive predictive value, *NPV* negative predictive value	

**Table 6:** Performance of cytology and HC2 in the follow-up of conservatively treated AIS

	HC2 (%)	Pap test (%)	Pap + HC2 (%)
First FU visit			
Sensitivity	90	60	90
Specificity	58	69	50
PPV	64	55	52
NPV	88	73	89
Second FU visit			
Sensitivity	84	66	100
Specificity	59	73	52
PPV	42	44	40
NPV	91	87	100
*HC2* Hybrid Capture 2, *AIS* Adenocarcinoma in situ, *FU* follow up, *PPV* positive predictive value, *NPV* negative predictive value			

**Table 7:** Minimum set of information on human papillomavirus for women

Basic notions—simple and comprehensible but scientifically correct—on:
cervical carcinoma and its precursors
the existence and the role of the human papilloma virus
aims of the screening strategy
the early diagnosis of the disease
Quantification of the problem, individualized and in the clearest of terms (personalization of the epidemiological data)
Clarification of some sensitive and potentially problematic concepts:
sexual transmissibility of HPV
widespread occurrence of the virus
behavioral implications correlated to the diagnosis
partner’s role and implications for the couple
substantial harmlessness of the transient infection
absence of specific anti-viral therapies
concepts of persistence, re-infection
